# Causal insights into how NAFLD progression drives abdominal aortic aneurysm: A bidirectional MR study integrating genetic and multi-omics profiling

**DOI:** 10.1097/MD.0000000000048613

**Published:** 2026-05-08

**Authors:** Rongyang Xi, Jiuqing Yan, Yu Wang, Lexi Li, Yongshun Liang, Siying Cai, Yijingxuan Duan

**Affiliations:** a The First School of Clinical Medicine, Nanfang Hospital, Southern Medical University, Guangzhou, Guangdong, China; b The Second School of Clinical Medicine, Zhujiang Hospital, Southern Medical University, Guangzhou, Guangdong, China; c Department of Biostatistics, School of Public Health, Southern Medical University, Guangzhou, Guangdong, China.

**Keywords:** abdominal aortic aneurysm, biomarkers, Mendelian randomization, multi-omics, nonalcoholic fatty liver disease

## Abstract

A well-defined causal relationship between nonalcoholic fatty liver disease (NAFLD) and abdominal aortic aneurysm (AAA) is lacking, and it remains unknown whether AAA risk varies across different NAFLD stages. This study applied Mendelian randomization (MR) to evaluate the stratified effects of different NAFLD stages on AAA. Multi-omics data were integrated to identify potential mediating pathways. Large-scale genome-wide association study summary data were analyzed using inverse-variance weighted as the primary MR method. MR-Egger and weighted median MR methods were employed as complementary approaches. Sensitivity analyses were conducted using MR-PRESSO (Mendelian randomization pleiotropy residual sum and outlier), Cochran *Q* test, and MR-Egger testing. To further ensure the robustness of MR results, funnel plots, scatter plots and leave-one-out analyses were also constructed. Further analyses were conducted to assess the causal effects of NAFLD/NASH on thoracic aortic aneurysm (TAA) and aortic aneurysm (AA). Multi-omics integration helped identify intermediate molecular traits. Bidirectional MR analysis demonstrated a positive causal effect of NAFLD/NASH on AAA risk (odds ratio = 1.05, 95% confidence interval: 1.01–1.09; *P* = .017), with no evidence for reverse causation. Validation analyses showed a reverse causal association between liver fat content and AAA, while other associations were nonsignificant. Further MR analyses revealed no significant causal effects of NAFLD/NASH on thoracic aortic aneurysm or unclassified aortic aneurysm. Multi-omics integration identified 1 metabolite and 2 lipid species associated with NAFLD/NASH; however, none directly caused AAA. NAFLD/NASH exerts a positive causal effect on AAA, predominantly in advanced disease stages such as NASH and fibrosis. Multi-omics evidence suggests that certain metabolites and lipid species may serve as biomarkers or indirectly promote AAA by driving NAFLD progression, influencing AAA risk indirectly through promoting progression of NAFLD/NASH. These findings provide genetic evidence for NAFLD-driven AAA pathogenesis, underscoring the importance of risk stratification and early intervention in advanced NAFLD.

## 1. Introduction

Abdominal aortic aneurysm (AAA) is a serious and potentially fatal vascular condition characterized by focal dilation of the abdominal aorta, with clinical diagnosis confirmed by imaging evidence of a maximum aortic diameter ≥ 30 mm.^[1]^ In 2019, the global prevalence of AAA among individuals aged 30 to 79 years was 0.92%, corresponding to 35.12 million cases.^[2]^ The high incidence and mortality risk of AAA underscore the importance of early screening.

Nonalcoholic fatty liver disease (NAFLD) has become one of the most common causes of chronic liver disease, with a prevalence of approximately 30% in adults.^[3,4]^ NAFLD is a disease continuum that progresses from steatosis to nonalcoholic steatohepatitis (NASH), liver fibrosis, cirrhosis, and ultimately hepatocellular carcinoma. Although numerous studies have confirmed a strong association between NAFLD and cardiovascular disease risk, research exploring the genetic relationship between NAFLD and AAA remains extremely limited.^[5,6]^ Consequently, it remains unclear whether NAFL, NASH, and cirrhosis exert stratified effects on AAA risk.

The potential link between NAFLD and AAA progression is multifactorial. A multicenter retrospective study conducted by Wang et al identified both NAFLD and maximum AAA diameter as independent predictors of AAA progression.^[7]^ Furthermore, previous studies indicate that while metabolic syndrome is a primary driver in the pathogenesis of many NAFLD cases, the disease also involves complex and dynamic heterogeneous interactions among various factors, including imbalances in adipokines and cytokines, as well as abnormalities in metabolites such as lipids, amino acids, and energy-related intermediates.^[8]^ Notably, some of these mechanistic factors have also been implicated in the progression of AAA.^[9]^ These overlapping mechanisms suggest a potential association between the development and progression of NAFLD and AAA.

Mendelian randomization (MR) provides a robust approach to address confounding biases inherent in traditional observational studies. While MR has been widely applied in the field of cardiovascular and metabolic diseases, its use in evaluating the association between different stages of NAFLD and cardiovascular conditions remains limited.^[10,11]^ In particular, the causal relationship between NAFLD and AAA has not been systematically investigated. Given the complexity of the mechanisms underlying the association between NAFLD and AAA, a simplified perspective is likely insufficient to elucidate their potential links.

Therefore, this study employs bidirectional MR analysis to evaluate the causal relationships between NAFLD/NASH and AAA. Furthermore, we integrated lipidomic and metabolomic data to explore whether specific molecular features contribute to AAA pathogenesis by driving the onset and progression of NAFLD/NASH. This approach aims to identify and prioritize candidate intermediate molecular traits that may underlie or mediate the causal link, thereby providing supportive evidence for clinical risk prediction and targeted intervention in AAA.

## 2. Methods

### 2.1. Data source

Genome-wide association study (GWAS) data for all diseases were obtained from the same ethnic group across different databases.^[12]^ NAFLD/NASH data came from the study by Anstee et al,^[13]^ and AAA data were derived from summary statistics in the Global Biobank.^[14]^ Two-sample MR analyses were performed using liver fat measurement data (from UK Biobank summary statistics) and NAFL data (from deCODE summary statistics) in relation to AAA.^[15,16]^ Additionally, forward MR analyses between NAFLD/NASH and TAA (data from FinnGen R12) as well as AA (data from FinnGen R12) were conducted as supplementary validation.^[17]^ Multi-omics MR analyses involving 179 lipid species and 233 metabolites in relation to both NAFLD and AAA were performed to further elucidate mediating pathways in AAA pathogenesis.^[18,19]^ All data sources are detailed in Table 1.

**Table 1:** Detailed information on genome-wide association study summary statistics used in the study.

Exposure	Outcome	Number of SNPs	MR-Egger intercept_*P*val	Cochran *Q*_*P*val (IVW)	MR-PRESSO global test_*P*val
NAFLD/NASH	AAA	21	0.731	0.768	0.764
NAFL	AAA	8	0.650	0.878	0.906
Liver fat measurement	AAA	5	0.288	0.049	0.156
AAA	NAFLD/NASH	12	0.883	0.063	0.185
AAA	NAFL	12	0.930	0.457	0.470
AAA	Liver fat measurement	13	0.188	0.574	0.576

AAA = abdominal aortic aneurysm, MR-Egger = Mendelian randomization-Egger, MR-PRESSO = Mendelian randomization pleiotropy residual sum and outlier, NAFL = nonalcoholic fatty liver, NAFLD = nonalcoholic fatty liver disease, NASH = nonalcoholic steatohepatitis, SNP = single nucleotide polymorphism.

### 2.2. Study design

**Figure 1.**
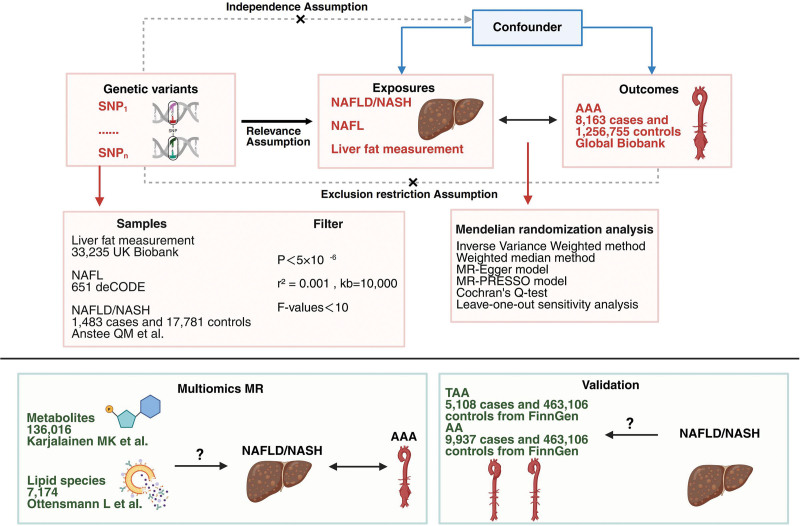
Study design and core assumptions of MR. Assumptions 1, 2, and 3 correspond to the relevance assumption, the independence assumption, and the exclusion restriction assumption, respectively (created with BioRender). AA = aortic aneurysm, AAA = abdominal aortic aneurysm, MR = Mendelian randomization, MR-PRESSO = Mendelian randomization pleiotropy residual sum and outlier test, NAFL = nonalcoholic fatty liver, NAFLD = nonalcoholic fatty liver disease, NASH = nonalcoholic steatohepatitis, TAA = thoracic aortic aneurysm, SNP = single nucleotide polymorphism.

This study employed a 2-sample bidirectional MR approach to investigate the causal relationships of NAFLD/NASH and its sub-phenotypes (including NAFL and liver fat content) with AAA. This design aimed to elucidate the differential effects of NAFLD progression stages and liver fat accumulation on AAA development. To examine whether the observed causal association between NAFLD/NASH and AAA is specific to its large-aneurysm pathology, we also performed forward MR analyses to confirm causal links between NAFLD/NASH and both TAA and AA, thereby ensuring comprehensiveness in our validation. Furthermore, it remains unclear whether the effects of multi-omics molecular features – such as alterations in lipid species and metabolites – on AAA are independent of NAFLD/NASH. Therefore, we integrated lipidomic and metabolomic data to conduct multi-omics MR analyses, seeking to identify molecules that may indirectly promote AAA initiation and progression by driving NAFLD/NASH. The overall study design is summarized in Figure 1.

### 2.3. Selection of instrumental variables

In the primary forward MR analysis (NAFLD/NASH → AAA), SNPs significantly associated with NAFLD at a genome-wide significance threshold of *P* < 5 × 10^−6^ were selected as instrumental variables (IVs). SNPs in linkage disequilibrium (LD) were removed (*r*^2^ > 0.001, clump distance < 10,000 kb). The *F*-statistic was calculated for each SNP (*F* = β^2^/SE^2^), and only strong IVs (*F* > 10) were retained. During harmonization, ambiguous and palindromic IVs were excluded, and no proxy SNPs were used. Eligible SNPs were included after removing those associated with susceptibility genes linked to impaired very low-density lipoprotein secretion.^[20]^ In validation analyses, the SNP selection threshold was *P* < 5 × 10^−6^ for NAFL and *P* < 5 × 10^−8^ for liver fat measurement, with other criteria unchanged. For reverse MR, AAA-associated SNPs (*P* < 5 × 10^−8^) were selected as IVs for the exposure, with NAFLD progression stages and liver fat as outcomes. Subsequent filtering steps mirrored those described above. Finally, in multi-omics MR analyses, only strong IVs meeting a genome-wide significance threshold of *P* < 1 × 10^−5^ and without evidence of LD were included. All IVs are presented in Tables S1,S2,S3,S4,S5,S6,S7,S8,S9 and S10, Supplemental Digital Content. For SNPs for which the rsID is not directly provided in public databases or literature, this study first performs a standardized conversion of the missing rsIDs by comparing them to genomic coordinates. If automatic matching is not possible, manual comparison and verification are conducted by referring to the variant annotation files from the original GWAS datasets, and the corresponding rsID information is manually supplemented. Except for the entries that can be traced and corrected, the remaining data that cannot be recovered are left missing and are not imputed to avoid introducing systematic bias.

### 2.4. Data analysis

When all selected SNPs are valid instrumental variables, the IVW method provides the most precise causal effect estimates. Additionally, this study employed the weighted median (WM) method and MR-Egger method as supplementary analyses. The WM method produces stable causal estimates when more than half of the SNPs are valid IVs by calculating the median of the distribution function of all SNP effect values sorted by weight.^[21]^ Meanwhile, the MR-Egger method provides reliable causal estimates even when all SNPs are considered invalid IVs. To assess the stability and reliability of Mendelian randomization results, multiple sensitivity analyses were implemented in this study. MR-PRESSO is a method for detecting and correcting outliers in IVW linear regression. If MR-PRESSO analysis detects significant pleiotropy (*P*-value below the MR-PRESSO outlier threshold), the MR analysis is repeated after removing the outlier SNP. Cochran *Q* test was used to assess the variability in individual genetic variance estimates. A Cochran *Q* test *P*-value > .05 indicated no heterogeneity. To detect potential pleiotropy, we also employed the MR-Egger test, where a *P*-value > .05 signaled no significant pleiotropy.^[22]^ To further ensure the robustness of MR results, funnel plots, scatter plots and leave-one-out analyses were constructed. These approaches were used to evaluate the robustness of the associations and to assess horizontal pleiotropy. In interpreting the results, the IVW estimate is prioritized as the primary analysis due to its highest statistical power under the assumption of valid instrumental variables. The weighted median and MR-Egger methods serve as sensitivity analyses to assess robustness, acknowledging that their nonsignificance may reflect lower statistical power, particularly for modest effect sizes.^[21]^ The strength of associations between various NAFLD stages and AAA, TAA, and AA was expressed as β values with 95% confidence intervals. To account for multiple testing across 3 comparisons related to different NAFLD stages and aortic aneurysm outcomes, a Bonferroni-corrected significance threshold was set at *P* < .017. Associations with *P* ≤ .05 but >0.017 were considered indicative of a potential causal relationship.^[23]^ All analyses were performed in R (version 4.4) using the “Two Sample MR,” “MR-PRESSO,” and “Forest Plot” packages.

## 3. Results

### 3.1. Bidirectional Mendelian randomization analysis between NAFLD/NASH and AAA

**Figure 2.**
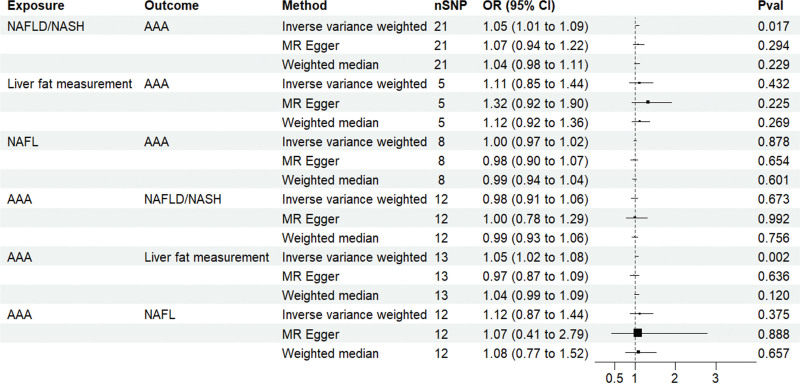
Bidirectional MR analysis evaluating the causal relationships between NAFLD/NASH, NAFL, liver fat content, and AAA. AAA = abdominal aortic aneurysm, CI = confidence interval, MR = Mendelian randomization, NAFL = nonalcoholic fatty liver, NAFLD = nonalcoholic fatty liver disease, NASH = nonalcoholic steatohepatitis, OR = odds ratio, SNP = single nucleotide polymorphism.

**Figure 3.**
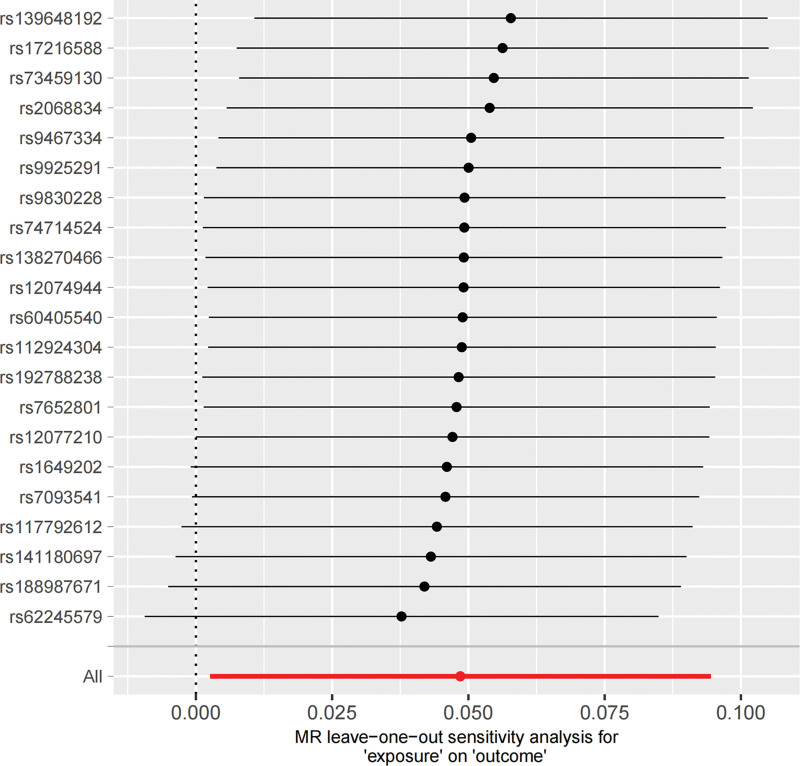
The bidirectional MR analyses: Plots of “leave-one-out” analyses for MR analyses of the causal effect of NAFLD/NASH with the risk of AAA. The horizontal lines in the figure represents β value and its 95% confidence interval (CI) of causal inference, which indicates the genetic effect of the SNP on cardiovascular disease. AAA = abdominal aortic aneurysm, CI = confidence interval, MR = Mendelian randomization, NAFLD = nonalcoholic fatty liver disease, NASH = nonalcoholic steatohepatitis, SNP = single nucleotide polymorphism.

**Figure 4.**
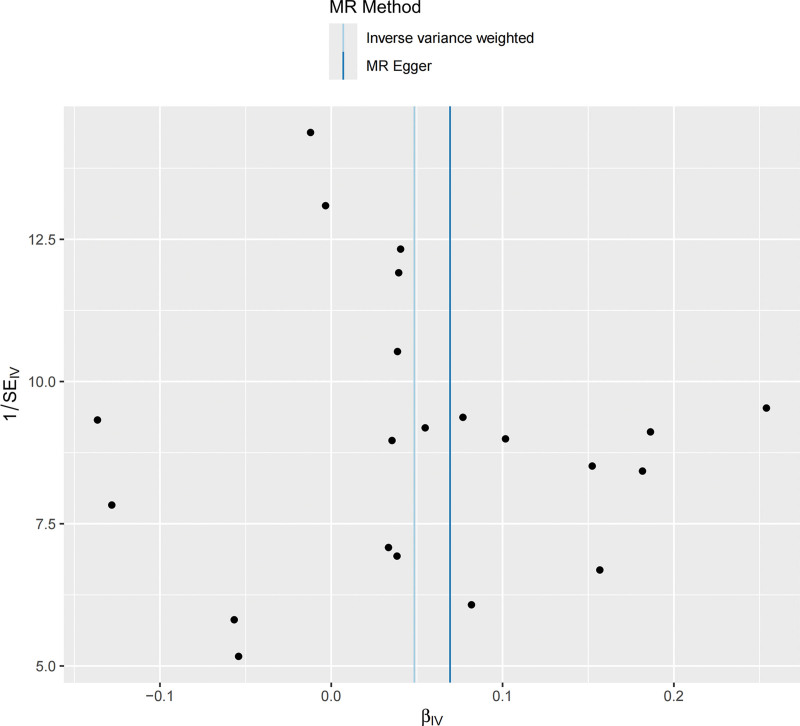
Funnel plot of the causal relationship between NAFLD/NASH and AAA, primarily evaluated using the IVW method. AAA = abdominal aortic aneurysm, MR = Mendelian randomization, NAFLD = nonalcoholic fatty liver disease, NASH = nonalcoholic steatohepatitis, SE = standard error.

NAFLD/NASH exhibited a significant causal effect on AAA (OR = 1.05, 95% CI: 1.01–1.09; *P* = .017). Although the results from MR-Egger (OR = 1.072, 95% CI: 0.94–1.22; *P* = .294) and weighted median methods (OR = 1.04, 95% CI: 0.98–1.11; *P* = .229) were not statistically significant, they demonstrated consistent directional trends (Fig. 2). The concordant positive direction across all methods, despite variation in statistical significance, provides additional support for the robustness of the observed causal effect rather than indicating random noise. Reverse MR analysis yielded *P*-values > .05, indicating no significant causal association between AAA and NAFLD/NASH (Fig. 2). The stability of the causal estimate from NAFLD/NASH to AAA is illustrated by the leave-one-out sensitivity analysis (Fig. 3). The statistical conclusion is strongly supported by the funnel plot, in which the effect estimates of individual genetic instruments are symmetrically distributed around the IVW pooled estimate, showing no evident directional skew (Fig. 4). Sensitivity analyses conducted in both directions showed no heterogeneity among instrumental variables via Cochran *Q* test, no horizontal pleiotropy per MR-Egger intercept test, and MR-PRESSO analysis confirmed the absence of horizontal pleiotropy (Table 2).

**Table 2:** Results of bidirectional Mendelian randomization sensitivity analyses between NAFLD/NASH, NAFL, liver fat measurement, and AAA.

Phenotypes	Consortium/Authors	Ethnicity	Sample size	Year	Data source
NAFLD/NASH	Anstee QM et al	European	1483 cases and 17,781 controls	2020	https://www.ebi.ac.uk/gwas/publications/32298765
NAFL	deCODE	European	651	2022	https://www.decode.com/summarydata/
AAA	Global Biobank	European	8163 cases and 12,56,755 controls	2022	https://www.ebi.ac.uk/gwas/studies/GCST90399672
TAA	FinnGen	European	5108 cases and 4,63,106 controls	2024	https://r12.finngen.fi/pheno/I9_THAORTANEUR
AA	FinnGen	European	9937 cases and 4,63,106 controls	2024	https://r12.finngen.fi/pheno/I9_AORTANEUR
Liver fat measurement	UK Biobank	European	33,235	2022	https://www.ebi.ac.uk/gwas/studies/GCST90267352
179 lipid species	Ottensmann L et al	European	7174	2023	https://pubmed.ncbi.nlm.nih.gov/37907536/
233 metabolites	Karjalainen MK et al	European	1,36,016	2024	https://pubmed.ncbi.nlm.nih.gov/38448586/

AA = aortic aneurysm, AAA = abdominal aortic aneurysm, MR = Mendelian randomization, NAFL = nonalcoholic fatty liver, NAFLD = nonalcoholic fatty liver disease, NASH = nonalcoholic steatohepatitis, TAA = thoracic aortic aneurysm.

### 3.2. Validation analysis of the causal effect of NAFLD/NASH on TAA and AA

According to the IVW method, no significant association was observed between NAFLD/NASH and either TAA or AA. The results were as follows: TAA (OR = 1.073, 95% CI: 0.94–1.23; *P* = .276), AA (OR = 1.04, 95% CI: 1.00–1.09; *P* = .052; Table 3). The stability of these causal estimates is illustrated by leave-one-out analyses (Fig. S1, Supplemental Digital Content). Sensitivity analysis for both outcomes revealed no heterogeneity among instrumental variables via Cochran *Q* test, no horizontal pleiotropy based on the MR-Egger intercept test, and MR-PRESSO analysis confirmed the absence of horizontal pleiotropy (Table 4).

**Table 3:** Estimates of genetical liability in Mendelian randomization.

Exposure	Outcome	Method	SNPs (n)	*P* value	OR (95% CI)
NAFLD/NASH	TAA	MR-Egger	23	.276	1.028 (0.978–1.081)
		Weighted median	23	.313	1.073 (0.939–1.227)
		Inverse variance weighted	23	.181	1.051 (0.977–1.131)
NAFLD/NASH	AA	MR-Egger	23	.052	1.044 (1.000–1.091)
		Weighted median	23	.189	1.084 (0.965–1.219)
		Inverse variance weighted	23	.137	1.042 (0.987–1.100)
3-hydroxybutyrate levels	NAFLD/NASH	MR-Egger	30	.001	0.479 (0.307–0.749)
		Weighted median	30	.021	0.434 (0.213–0.881)
		Inverse variance weighted	30	.019	0.434 (0.213–0.881)
Phosphatidylcholine (18:1_20:4) levels	NAFLD/NASH	MR-Egger	21	.308	1.315 (0.788–2.195)
		Weighted median	21	.149	1.240 (0.926–1.661)
		Inverse variance weighted	21	.011	1.282 (1.057–1.556)
Sphingomyelin (d36:2) levels	NAFLD/NASH	MR-Egger	18	.270	1.277 (0.839–1.944)
		Weighted median	18	.124	1.214 (0.948–1.554)
		Inverse variance weighted	18	.014	1.240 (1.044–1.472)

AA = aortic aneurysm, CI = confidence interval, MR-Egger = Mendelian randomization-Egger, NAFLD = nonalcoholic fatty liver disease, NASH = nonalcoholic steatohepatitis, OR = odds ratio, TAA = thoracic aortic aneurysm.

**Table 4:** Sensitivity analyses in Mendelian randomization.

Exposure	Outcome	SNPs (n)	MR-Egger intercept_*P*val	Cochran *Q*_*P*val (IVW)	MR-PRESSO global test_*P*val
NAFLD/NASH	TAA	23	0.506	0.443	0.468
	AA	23	0.504	0.082	0.078
3-hydroxybutyrate levels	NAFLD/NASH	30	0.213	0.636	0.657
Phosphatidylcholine (18:1_20:4) levels	NAFLD/NASH	21	0.917	0.433	0.48
Sphingomyelin (d36:2) levels	NAFLD/NASH	18	0.88	0.742	0.742

AA = aortic aneurysm, AAA = abdominal aortic aneurysm, IVW = inverse-variance weighted, MR-Egger = Mendelian randomization-Egger, MR-PRESSO = Mendelian randomization pleiotropy residual sum and outlier, NAFLD = nonalcoholic fatty liver disease, NASH = nonalcoholic steatohepatitis, SNP = single nucleotide polymorphism, TAA = thoracic aortic aneurysm.

### 3.3. Bidirectional Mendelian randomization analysis of liver fat measurement, NAFL, and AAA

Forward MR analysis revealed no significant causal effect of either liver fat content or NAFL on AAA (Fig. 2). However, reverse MR analysis revealed a significant causal effect of AAA on liver fat content (*P* = .010), while no significant causal relationship was observed between AAA and NAFL. Leave-one-out analyses of liver fat content and NAFL in relation to AAA are shown in Figures S2 and S3, Supplemental Digital Content, respectively. Cochran *Q* test indicated minimal heterogeneity in the forward MR estimates between liver fat content and AAA, while no heterogeneity was detected in the remaining analyses. Both MR-Egger intercept tests and MR-PRESSO analysis confirmed the absence of horizontal pleiotropy (Table 2).

### 3.4. Multi-omics MR analysis of lipid species, metabolites, and NAFLD/NASH: a forward Mendelian randomization study

In the MR analysis of 233 metabolites and NAFLD/NASH, initial screening identified 5 metabolites significantly associated with NAFLD/NASH. After removing outlier SNPs using MR-PRESSO, only the level of 3-hydroxybutyrate was found to be inversely associated with the risk of NAFLD/NASH (IVW: OR = 0.43, 95% CI: 0.21–0.88; *P* = .019; Table 3), suggesting it may serve as a protective factor. Following outlier removal, Cochran *Q* test, MR-Egger intercept analysis, and MR-PRESSO indicated no evidence of horizontal pleiotropy or heterogeneity (Table 4). Inverse-variance weighted MR analysis further revealed that elevated levels of serum phosphatidylcholine (18:1–20:4; OR = 1.28, 95% CI: 1.06–1.56; *P* = .011) and serum sphingomyelin (d36:2; OR = 1.24, 95% CI: 1.04–1.47; *P* = .014) were positively associated with NAFLD/NASH (Table 3), indicating these lipid species may increase NAFLD/NASH risk. No lipid species showed a significant inverse association with NAFLD/NASH. Sensitivity analyses yielded consistent results, with no anomalies detected (Table 4). The leave-one-out sensitivity analyses for these metabolites and lipids in relation to NAFLD/NASH are presented in Figure S4, Supplemental Digital Content.

### 3.5. Multi-omics Mendelian randomization analysis of the causal effects of lipid species and metabolites on AAA

No significant causal relationship was identified between NAFLD/NASH-associated lipid species or metabolites and AAA after the removal of outlier SNPs via MR-PRESSO. Sensitivity analyses, including Cochran *Q* test, MR-Egger intercept test, and MR-PRESSO, were conducted, and all returned negative results.

## 4. Discussion

To our knowledge, this is the first study to systematically investigate the potential causal relationship between NAFLD/NASH and AAA using MR. The main findings indicate a significant positive causal effect of NAFLD/NASH on AAA, while no evidence supports a reverse causal direction. This conclusion is based on the significant IVW result after confirming the core MR assumptions of no heterogeneity or horizontal pleiotropy. The interpretation that prioritizes the IVW estimate over nonsignificant sensitivity analyses (due to their lower power) aligns with established standards in the MR literature, as seen in studies of cardiovascular and liver diseases.^[24,25]^

The conclusions of this study are supported by previous clinical research. A multicenter retrospective study, which included AAA patients and healthy controls who underwent abdominal CTA and non-contrast CT, demonstrated that NAFLD detected via non-contrast CT served as an independent predictor of AAA progression.^[7]^ However, this study primarily reflects an association between the overall status of NAFLD and AAA progression, rather than elucidating the differential impacts of various NAFLD stages on AAA risk. It is noteworthy that the metabolic and inflammatory burden varies across different pathological stages of NAFLD. Patients with advanced disease often exhibit insulin resistance and systemic inflammatory responses, both of which are established independent risk factors for cardiovascular diseases, including AAA.^[26]^ In contrast, individuals with simple steatosis generally present with milder metabolic disturbances. It can thus be hypothesized that the significant risk association between NAFLD and AAA observed in previous clinical studies may largely reflect the effect of metabolic dysregulation in advanced stages, rather than a causal role of NAFLD as a uniform entity.

Based on these considerations, this study stratified NAFLD by disease stage and employed a 2-sample MR approach for validation. The results demonstrated no significant bidirectional causal relationship between NAFL and AAA. In contrast, a positive causal effect of NAFLD/NASH (a cohort in which 56.4% had NASH and 70.7% had fibrosis) on AAA was observed.^[13]^ These findings suggest that the potential pathogenic effect of NAFLD on AAA is primarily concentrated in the advanced stages rather than the simple steatosis phase. Supporting this conclusion, a recent prospective cohort study using liver biopsy to determine fibrosis severity reported that among NAFLD patients without a history of cardiovascular disease, advanced liver fibrosis (stages F3–F4) was an independent predictor of future cardiovascular events (adjusted SHR = 2.86, 95% CI: 1.36–6.04). In contrast, patients with simple steatosis without significant fibrosis did not exhibit a markedly increased cardiovascular risk.^[27]^ Furthermore, evidence from the UK Biobank prospective cohort reinforced the association between metabolic dysfunction-associated fatty liver disease (MAFLD) and AAA. The results indicated that MAFLD significantly increased the risk of AAA (OR = 1.52, 95% CI: 1.35–1.71, *P* < .001), with the risk escalating in accordance with the severity of fibrosis scores in MAFLD. It is noteworthy that MAFLD and NAFLD refer to the same disease entity with different diagnostic criteria, both centered on fatty liver disease accompanied by metabolic dysregulation.^[28]^

Interestingly, reverse MR analysis in this study revealed a significant causal effect of AAA on liver fat content, but no genetic association with NAFLD/NASH. NAFLD/NASH represents an advanced stage of hepatic steatosis driven by inflammatory responses and fibrosis; in contrast, liver fat content reflects only the extent of fat deposition itself. It can thus be inferred that AAA may genetically contribute to liver fat accumulation without necessarily driving progressive NAFLD/NASH. A previous observational study reporting an elevated risk of NAFLD/NASH in AAA patients may have arisen from a failure to distinguish between simple steatosis and progressive NAFLD/NASH.^[29]^ In essence, our stratified MR results provide more refined and compelling causal evidence regarding these distinct relationships.

Based on the analytical results of this study, NAFL did not demonstrate a significant genetic causal effect on AAA in the general population, whereas NAFLD/NASH was significantly associated with an increased risk of AAA. This finding carries important clinical implications. In the management of patients with NAFL without significant fibrosis or inflammatory progression, clinicians may primarily focus on controlling metabolic abnormalities and implementing lifestyle interventions to reduce the risk of disease progression. For patients who have advanced to NASH or present with fibrosis, AAA risk should be incorporated into routine monitoring and comprehensive management, including aortic evaluation using imaging modalities. On the other hand, our reverse MR analysis suggests that AAA patients are more susceptible to liver fat accumulation. This indicates that clinical management of AAA should include attention to abnormal liver fat metabolism, enabling early identification and intervention to prevent overlapping disease risks. Notably, recent studies have shown that microRNA-33 plays a key regulatory role in lipid metabolism and energy homeostasis. Its deficiency ameliorates multiple metabolic-cardiovascular phenotypes – including NAFLD and AAA – in animal models, suggesting it as a potential target for preventing and treating both conditions.^[30]^

In recent years, emerging studies have suggested that multi-omics molecular features may act as candidate intermediate molecular traits mediating the pathogenesis of AAA.^[31,32]^ However, whether they influence AAA independently or via the mediation of NAFLD/NASH remains unclear. To further elucidate this causal pathway, this study employed multi-omics MR analysis, leveraging lipidomic and metabolomic data to investigate whether these molecular features contribute to AAA initiation and progression by driving the development of NAFLD/NASH. At the metabolomic level, our analysis identified a significant inverse association between 3-hydroxybutyrate and the risk of NAFLD/NASH. 3-hydroxybutyrate, as a candidate intermediate molecular trait, participates in energy metabolism while exerting antioxidant and anti-inflammatory effects to alleviate mitochondrial dysfunction and inflammatory responses – key clues implicated in NAFLD progression.^[25]^ Thus, our findings provide genetic support for previous studies highlighting the protective role of ketone bodies in metabolic diseases. In the context of AAA pathogenesis, the protective effect of 3-hydroxybutyrate may indirectly reduce AAA risk by lowering the incidence of NAFLD/NASH. In the lipidomic profile, both serum phosphatidylcholine and sphingomyelin were significantly associated with NAFLD/NASH. Elevated levels may alter hepatocellular membrane composition, thereby activating inflammatory pathways and immune responses, and accelerating the pathological transition from steatosis to inflammation and fibrosis. Collectively, our results suggest that 3-hydroxybutyrate, phosphatidylcholine, and sphingomyelin influence AAA risk primarily through mediating NAFLD/NASH progression rather than via direct effects. These multi-omics MR findings indicate that NAFLD/NASH may serve as a central mediator between metabolic disturbances and AAA. In terms of clinical monitoring, the use of multi-omics biomarkers for risk stratification and follow-up holds promise for earlier identification of high-risk populations for AAA. Furthermore, regulating metabolic pathways – such as exogenous supplementation of 3-hydroxybutyrate or modulation of phosphatidylcholine and sphingolipid levels – may represent novel therapeutic strategies for delaying NAFLD progression and preventing AAA.

This study has several limitations. First, all GWAS data included were derived from European populations, which limits the generalizability and external validity of our findings to other ethnicities or geographic regions. Furthermore, this study did not explore the bidirectional causal relationship between NAFLD-derived cirrhosis and AAA, primarily due to the lack of high-quality GWAS data specifically targeting cirrhosis as a distinct subtype. Therefore, future research should focus more closely on the stratified genetic evidence of different pathological stages of NAFLD, combined with larger and more refined cohort data, in order to precisely identify key turning points in the disease progression. On this basis, multicenter, long-term follow-up randomized controlled trials should be promoted to verify the intervention effects of targeting metabolic pathways or anti-inflammatory treatments on the high-risk population for AAA. The ultimate goal is to develop a risk assessment model based on NAFLD/NASH, integrating multi-omics biomarkers, which will facilitate early identification and intervention of AAA across society, thereby reducing the disease burden and improving patients’ quality of life.

## 5. Conclusion

Through systematic genetic analyses, this study provides the first evidence of a significant positive causal effect of NAFLD/NASH on AAA, which appears to be driven primarily by advanced disease stages rather than simple steatosis. Multi-omics insights further indicate that certain metabolites and lipid species indirectly contribute to AAA risk by promoting the progression of NAFLD/NASH. These findings support incorporating NAFLD/NASH – particularly in its advanced stages – into early screening and monitoring strategies for populations at high risk for AAA. Moreover, the results indicate that modulating hepatic metabolism represents a promising preventive strategy against AAA.

## Author contributions

**Conceptualization:** Rongyang Xi, Jiuqing Yan.

**Data curation:** Rongyang Xi, Jiuqing Yan, Yu Wang, Lexi Li, Yongshun Liang, Siying Cai, Yijingxuan Duan.

**Formal analysis:** Rongyang Xi, Jiuqing Yan, Yu Wang, Lexi Li, Yongshun Liang, Siying Cai, Yijingxuan Duan.

**Investigation:** Rongyang Xi, Jiuqing Yan.

**Methodology:** Rongyang Xi, Jiuqing Yan.

**Project administration:** Rongyang Xi.

**Supervision:** Rongyang Xi.

**Visualization:** Rongyang Xi, Jiuqing Yan, Yu Wang, Lexi Li, Yongshun Liang, Siying Cai, Yijingxuan Duan.

**Writing – original draft:** Rongyang Xi, Jiuqing Yan.

**Writing – review & editing:** Rongyang Xi.



**Figure:**
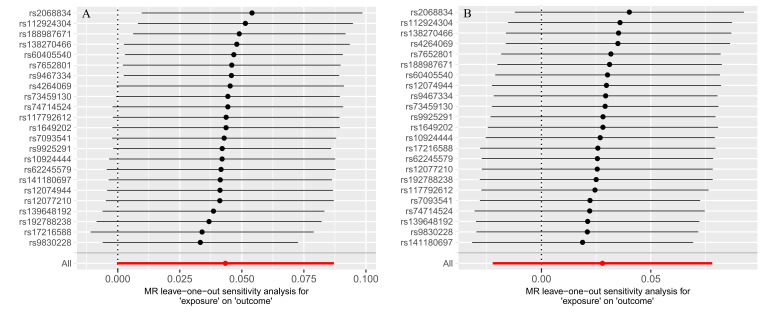


**Figure:**
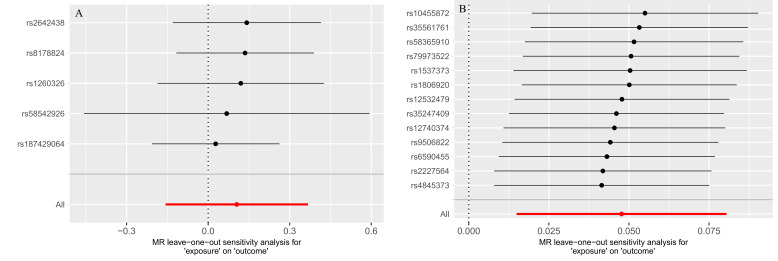


**Figure:**
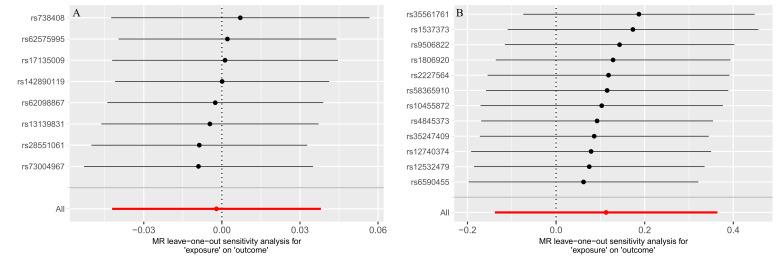


**Figure:**
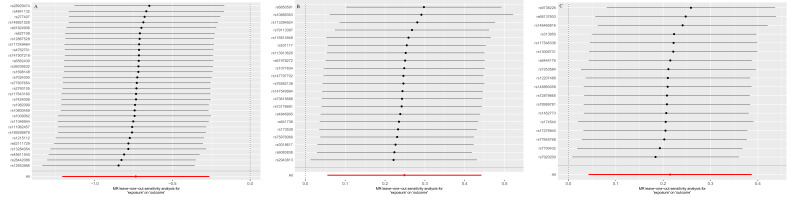




















## References

[1] Wanhainen A Van Herzeele I Bastos Goncalves F; ESVS Guidelines Committee. Editor’s Choice – European Society for Vascular Surgery (ESVS) 2024 clinical practice guidelines on the management of abdominal aorto-iliac artery aneurysms. Eur J Vasc Endovasc Surg. 2024;67:192–331. 38307694 10.1016/j.ejvs.2023.11.002

[2] Song P He Y Adeloye D; Global Health Epidemiology Research Group (GHERG). The global and regional prevalence of abdominal aortic aneurysms: a systematic review and modeling analysis. Ann Surg. 2023;277:912–9. 36177847 10.1097/SLA.0000000000005716 PMC10174099

[3] Powell EE Wong VW Rinella M. Non-alcoholic fatty liver disease. Lancet. 2021;397:2212–24. 33894145 10.1016/S0140-6736(20)32511-3

[4] Han SK Baik SK Kim MY. Non-alcoholic fatty liver disease: definition and subtypes. Clin Mol Hepatol. 2023;29(suppl):S5–S16. 36577427 10.3350/cmh.2022.0424 PMC10029964

[5] Zheng H Sechi LA Navarese EP Casu G Vidili G. Metabolic dysfunction-associated steatotic liver disease and cardiovascular risk: a comprehensive review. Cardiovasc Diabetol. 2024;23:346. 39342178 10.1186/s12933-024-02434-5 PMC11439309

[6] Sarikaya R Şengül C Kümet O İmre G Akbulut T Oğuz M. Fragmented QRS in inferior leads is associated with non-alcholic fatty liver disease, body-mass index, and interventricular septum thickness in young men. Anatol J Cardiol. 2022;26:100–4. 35190357 10.5152/AnatolJCardiol.2021.433 PMC8878927

[7] Wang X Sun J Chang N Liu M Zhang S. Association between non-alcoholic fatty liver disease and progression of abdominal aortic aneurysm: a multicenter study. BMC Med Imaging. 2025;25:24. 39833711 10.1186/s12880-025-01559-7 PMC11749205

[8] Grander C Grabherr F Tilg H. Non-alcoholic fatty liver disease: pathophysiological concepts and treatment options. Cardiovasc Res. 2023;119:1787–98. 37364164 10.1093/cvr/cvad095 PMC10405569

[9] Schanzer A Oderich GS. Management of abdominal aortic aneurysms. N Engl J Med. 2021;385:1690–8. 34706173 10.1056/NEJMcp2108504

[10] Lv Y Shen D Zhang G. Causal associations between the gut microbiome and aortic aneurysm: a Mendelian randomization study. Cardiovasc Innov Appl. 2024;9:44.

[11] Song Q Shang L Zhang Y Cui Y Du J Hou Y. Rheumatoid arthritis and risk of atrial fibrillation: results from pooled cohort studies and Mendelian randomization analysis. Cardiovasc Innov Appl. 2024;9:25.

[12] Kavousi M Bos MM Barnes HJ. Multi-ancestry genome-wide study identifies effector genes and druggable pathways for coronary artery calcification. Nat Genet. 2023;55:1651–64. 37770635 10.1038/s41588-023-01518-4 PMC10601987

[13] Anstee QM Darlay R Cockell S; EPoS Consortium Investigators. Genome-wide association study of non-alcoholic fatty liver and steatohepatitis in a histologically characterised cohort(☆). J Hepatol. 2020;73:505–15. 32298765 10.1016/j.jhep.2020.04.003

[14] Zhou W Kanai M Wu KH; Biobank of the Americas. Global Biobank meta-analysis initiative: powering genetic discovery across human disease. Cell Genom. 2022;2:100192. 36777996 10.1016/j.xgen.2022.100192 PMC9903716

[15] van der Meer D Gurholt TP Sønderby IE. The link between liver fat and cardiometabolic diseases is highlighted by genome-wide association study of MRI-derived measures of body composition. Commun Biol. 2022;5:1271. 36402844 10.1038/s42003-022-04237-4 PMC9675774

[16] Sveinbjornsson G Ulfarsson MO Thorolfsdottir RB; DBDS Genomic consortium. Multiomics study of nonalcoholic fatty liver disease. Nat Genet. 2022;54:1652–63. 36280732 10.1038/s41588-022-01199-5 PMC9649432

[17] Kurki MI Karjalainen J Palta P; FinnGen. FinnGen provides genetic insights from a well-phenotyped isolated population. Nature. 2023;613:508–18. 36653562 10.1038/s41586-022-05473-8 PMC9849126

[18] Ottensmann L Tabassum R Ruotsalainen SE; FinnGen. Genome-wide association analysis of plasma lipidome identifies 495 genetic associations. Nat Commun. 2023;14:6934. 37907536 10.1038/s41467-023-42532-8 PMC10618167

[19] Karjalainen MK Karthikeyan S Oliver-Williams C; China Kadoorie Biobank Collaborative Group. Genome-wide characterization of circulating metabolic biomarkers. Nature. 2024;628:130–8. 38448586 10.1038/s41586-024-07148-y PMC10990933

[20] Ren Z Simons PIHG Wesselius A Stehouwer CDA Brouwers MCGJ. Relationship between NAFLD and coronary artery disease: a Mendelian randomization study. Hepatology. 2023;77:230–8. 35441719 10.1002/hep.32534 PMC9970021

[21] Mounier N Kutalik Z. Bias correction for inverse variance weighting Mendelian randomization. Genet Epidemiol. 2023;47:314–31. 37036286 10.1002/gepi.22522

[22] Burgess S Thompson SG. Interpreting findings from Mendelian randomization using the MR-Egger method. Eur J Epidemiol. 2017;32:377–89. 28527048 10.1007/s10654-017-0255-x PMC5506233

[23] Armstrong RA. When to use the Bonferroni correction. Ophthalmic Physiol Opt. 2014;34:502–8. 24697967 10.1111/opo.12131

[24] Peng H Wang S Wang M. Nonalcoholic fatty liver disease and cardiovascular diseases: a Mendelian randomization study. Metabolism. 2022;133:155220. 35618017 10.1016/j.metabol.2022.155220

[25] Tang M Tu Y Gong Y. beta-hydroxybutyrate facilitates mitochondrial-derived vesicle biogenesis and improves mitochondrial functions. Mol Cell. 2025;85:1395–410.e5. 40118051 10.1016/j.molcel.2025.02.022

[26] Zheng S Tsao PS Pan C. Abdominal aortic aneurysm and cardiometabolic traits share strong genetic susceptibility to lipid metabolism and inflammation. Nat Commun. 2024;15:5652. 38969659 10.1038/s41467-024-49921-7 PMC11226445

[27] Henson JB Simon TG Kaplan A Osganian S Masia R Corey KE. Advanced fibrosis is associated with incident cardiovascular disease in patients with non-alcoholic fatty liver disease. Aliment Pharmacol Ther. 2020;51:728–36. 32043602 10.1111/apt.15660 PMC7069774

[28] Jia Y Li Y Yu J. Association between metabolic dysfunction-associated fatty liver disease and abdominal aortic aneurysm. Nutr Metab Cardiovasc Dis. 2024;34:953–62. 38161123 10.1016/j.numecd.2023.11.004

[29] Mahamid M Khoury T Mahamid B Sbeit W Mari A Nseir W. The interplay between abdominal aortic aneurysm, metabolic syndrome and fatty liver disease: a retrospective case-control study. Diabetes Metab Syndr Obes. 2019;12:1743–9. 31564942 10.2147/DMSO.S205568 PMC6732573

[30] Ortega R Liu B Persaud SJ. Effects of miR-33 deficiency on metabolic and cardiovascular diseases: implications for therapeutic intervention. Int J Mol Sci . 2023;24:10777. 37445956 10.3390/ijms241310777 PMC10342147

[31] Ferreira HB Trindade F Nogueira-Ferreira R. Lipidomic insights on abdominal aortic aneurysm and peripheral arterial disease. J Mol Med (Berl). 2025;103:365–80. 40011252 10.1007/s00109-025-02524-1 PMC12003574

[32] Lieberg J Wanhainen A Ottas A. Metabolomic profile of abdominal aortic aneurysm. Metabolites. 2021;11:555. 34436496 10.3390/metabo11080555 PMC8401627

