# Supplementary material for: Causal insights into how NAFLD progression drives abdominal aortic aneurysm: A bidirectional MR study integrating genetic and multi-omics profiling
Source: Medicine (Baltimore). 2026 May 8;105(19):e48613. doi: 10.1097/MD.0000000000048613 (PMC13166516; doi:10.1097/MD.0000000000048613)
Supplement: Supplementary file 1 [file medi-105-e48613-s001.doc]

Table S1. Instrumental variables used in MR analysis of the association between Liver fat measurement and AAA.

| Exposure | Outcome | SNP | Effect_allele | Other_allele | Exposure | | | | Outcome | | F |
| --- | --- | --- | --- | --- | --- | --- | --- | --- | --- | --- | --- |
|  |  |  |  |  | Beta | SE | pval | Beta | SE | pval |  |
| Liver fat measurement | AAA | rs1260326 | C | T | 0.056060625 | 0.007900244 | 1.28361E-12 | -0.005617 | 0.018488 | 0.7613 | 50.35410584 |
| Liver fat measurement | AAA | rs187429064 | A | G | 0.314375484 | 0.03484486 | 1.84437E-19 | 0.17398 | 0.070922 | 0.01416 | 81.39915453 |
| Liver fat measurement | AAA | rs2642438 | G | A | -0.064901859 | 0.008450176 | 1.58428E-14 | 0.023868 | 0.020867 | 0.2527 | 58.99058716 |
| Liver fat measurement | AAA | rs58542926 | C | T | 0.323801846 | 0.014525651 | 4.4451E-110 | 0.03981 | 0.034204 | 0.2445 | 496.9210926 |
| Liver fat measurement | AAA | rs8178824 | C | T | 0.135008235 | 0.023114381 | 5.1927E-09 | -0.087526 | 0.058855 | 0.137 | 34.11583245 |

AAA = abdominal aortic aneurysm, SNP = single nucleotide polymorphism.
