# Supplementary material for: Causal insights into how NAFLD progression drives abdominal aortic aneurysm: A bidirectional MR study integrating genetic and multi-omics profiling
Source: Medicine (Baltimore). 2026 May 8;105(19):e48613. doi: 10.1097/MD.0000000000048613 (PMC13166516; doi:10.1097/MD.0000000000048613)
Supplement: Supplementary file 2 [file medi-105-e48613-s006.doc]

Table S2. Instrumental variables used in MR analysis of the association between AAA and Liver fat measurement.

| Exposure | Outcome | SNP | Effect_allele | Other_allele | Exposure | | | Outcome | | | F |
| --- | --- | --- | --- | --- | --- | --- | --- | --- | --- | --- | --- |
|  |  |  |  |  | Beta | SE | pval | Beta | SE | pval |  |
| AAA | Liver fat measurement | rs10455872 | G | A | 0.3189 | 0.035182 | 1.255e-19 | 0.000918781784297212 | 0.0142158416059146 | 0.948467926321395 | 82.16143009 |
| AAA | Liver fat measurement | rs12532479 | C | T | 0.16069 | 0.027659 | 6.26e-09 | 0.00724371792993391 | 0.0126055493618599 | 0.565531302181917 | 33.75240728 |
| AAA | Liver fat measurement | rs12740374 | T | G | -0.17961 | 0.021705 | 1.285e-16 | -0.011991677 | 0.00928516176979143 | 0.196534454408661 | 68.47648281 |
| AAA | Liver fat measurement | rs1537373 | G | T | 0.20234 | 0.017944 | 1.727e-29 | 0.00735766308781636 | 0.00773429835876136 | 0.341450460867365 | 127.1525195 |
| AAA | Liver fat measurement | rs1806920 | A | G | 0.099259 | 0.018024 | 3.652e-08 | -0.000305364 | 0.007763455424264 | 0.968624438760415 | 30.32755742 |
| AAA | Liver fat measurement | rs2227564 | C | T | -0.13589 | 0.020306 | 2.203e-11 | -0.01762882 | 0.00881213553723884 | 0.0454445549529892 | 44.7843457 |
| AAA | Liver fat measurement | rs35247409 | T | C | -0.12817 | 0.022729 | 1.71e-08 | -0.010270208 | 0.0098816468923906 | 0.298655219490453 | 31.79890105 |
| AAA | Liver fat measurement | rs35561761 | T | C | 0.12762 | 0.021497 | 2.907e-09 | -0.004042371 | 0.0083459847979154 | 0.628138070671062 | 35.24372206 |
| AAA | Liver fat measurement | rs4845373 | T | C | -0.10324 | 0.018448 | 2.192e-08 | -0.017529881 | 0.00785230253428922 | 0.0255851728185093 | 31.31824461 |
| AAA | Liver fat measurement | rs58365910 | C | T | 0.13508 | 0.018956 | 1.032e-12 | -7.01E-05 | 0.00824775636953214 | 0.993219905321988 | 50.77953316 |
| AAA | Liver fat measurement | rs6590455 | C | T | 0.11442 | 0.020059 | 1.169e-08 | 0.0142751761676932 | 0.00808589680534445 | 0.077489627716399 | 32.53758608 |
| AAA | Liver fat measurement | rs79973522 | C | T | 0.1861 | 0.031935 | 5.631e-09 | 9.76943814203231e-05 | 0.0127853291649703 | 0.99390331845771 | 33.95931374 |
| AAA | Liver fat measurement | rs9506822 | G | A | -0.12948 | 0.021753 | 2.642e-09 | -0.013916767 | 0.00920468976185722 | 0.130553857777326 | 35.42966629 |

AAA = abdominal aortic aneurysm, SNP = single nucleotide polymorphism.
