# Supplementary material for: Causal insights into how NAFLD progression drives abdominal aortic aneurysm: A bidirectional MR study integrating genetic and multi-omics profiling
Source: Medicine (Baltimore). 2026 May 8;105(19):e48613. doi: 10.1097/MD.0000000000048613 (PMC13166516; doi:10.1097/MD.0000000000048613)
Supplement: Supplementary file 3 [file medi-105-e48613-s007.doc]

Table S3. Instrumental variables used in MR analysis of the association between NAFL and AAA.

| Exposure | Outcome | SNP | Effect_allele | Other_allele | Exposure | | | Outcome | | | F |
| --- | --- | --- | --- | --- | --- | --- | --- | --- | --- | --- | --- |
|  |  |  |  |  | Beta | SE | pval | Beta | SE | pval |  |
| NAFL | AAA | rs13139831 | A | G | 0.4704 | 0.103 | 4.95e-06 | 0.013415 | 0.034815 | 0.7 | 20.85740032 |
| NAFL | AAA | rs142890119 | G | A | 0.983 | 0.203 | 1.36e-06 | -0.04279 | 0.090124 | 0.6349 | 23.44849426 |
| NAFL | AAA | rs17135009 | C | T | 1.042 | 0.211 | 8.26e-07 | -0.022264 | 0.055867 | 0.6903 | 24.38768222 |
| NAFL | AAA | rs28551061 | A | G | -0.2645 | 0.0564 | 2.74e-06 | -0.024894 | 0.021342 | 0.2434 | 21.99343909 |
| NAFL | AAA | rs62098867 | G | A | 0.8229 | 0.175 | 2.75e-06 | 0.0037735 | 0.065812 | 0.9543 | 22.11149094 |
| NAFL | AAA | rs62575995 | A | G | 0.2683 | 0.0566 | 2.13e-06 | -0.01322 | 0.019198 | 0.4911 | 22.47027994 |
| NAFL | AAA | rs73004967 | A | G | 0.7179 | 0.123 | 5.8e-09 | 0.022785 | 0.035627 | 0.5225 | 34.06572873 |
| NAFL | AAA | rs738408 | C | T | 0.6537 | 0.0708 | 2.63e-20 | -0.012588 | 0.022682 | 0.5789 | 85.24922795 |

NAFL=non-alcoholic fatty Liver, AAA = abdominal aortic aneurysm, SNP = single nucleotide polymorphism.
