# Supplementary material for: Causal insights into how NAFLD progression drives abdominal aortic aneurysm: A bidirectional MR study integrating genetic and multi-omics profiling
Source: Medicine (Baltimore). 2026 May 8;105(19):e48613. doi: 10.1097/MD.0000000000048613 (PMC13166516; doi:10.1097/MD.0000000000048613)
Supplement: Supplementary file 4 [file medi-105-e48613-s008.doc]

Table S4. Instrumental variables used in MR analysis of the association between AAA and NAFL.

| Exposure | Outcome | SNP | Effect_allele | Other_allele | Exposure | | | Outcome | | | F |
| --- | --- | --- | --- | --- | --- | --- | --- | --- | --- | --- | --- |
|  |  |  |  |  | Beta | SE | pval | Beta | SE | pval |  |
| AAA | NAFL | rs10455872 | G | A | 0.3189 | 0.035182 | 1.255e-19 | 0.0672 | 0.135 | 0.618 | 82.16143009 |
| AAA | NAFL | rs12532479 | C | T | 0.16069 | 0.027659 | 6.26e-09 | 0.1007 | 0.0785 | 0.2 | 33.75240728 |
| AAA | NAFL | rs12740374 | T | G | -0.17961 | 0.021705 | 1.285e-16 | -0.0672 | 0.0679 | 0.322 | 68.47648281 |
| AAA | NAFL | rs1537373 | G | T | 0.20234 | 0.017944 | 1.727e-29 | -0.0229 | 0.0564 | 0.685 | 127.1525195 |
| AAA | NAFL | rs1806920 | A | G | 0.099259 | 0.018024 | 3.652e-08 | -0.0184 | 0.0568 | 0.746 | 30.32755742 |
| AAA | NAFL | rs2227564 | C | T | -0.13589 | 0.020306 | 2.203e-11 | -0.0072 | 0.06 | 0.904 | 44.7843457 |
| AAA | NAFL | rs35247409 | T | C | -0.12817 | 0.022729 | 1.71e-08 | -0.0777 | 0.0723 | 0.283 | 31.79890105 |
| AAA | NAFL | rs35561761 | T | C | 0.12762 | 0.021497 | 2.907e-09 | -0.1021 | 0.0597 | 0.0872 | 35.24372206 |
| AAA | NAFL | rs4845373 | T | C | -0.10324 | 0.018448 | 2.192e-08 | -0.0537 | 0.0606 | 0.376 | 31.31824461 |
| AAA | NAFL | rs58365910 | C | T | 0.13508 | 0.018956 | 1.032e-12 | 0.0119 | 0.0589 | 0.84 | 50.77953316 |
| AAA | NAFL | rs6590455 | C | T | 0.11442 | 0.020059 | 1.169e-08 | 0.105 | 0.0601 | 0.0806 | 32.53758608 |
| AAA | NAFL | rs9506822 | G | A | -0.12948 | 0.021753 | 2.642e-09 | 0.0481 | 0.0682 | 0.481 | 35.42966629 |

AAA = abdominal aortic aneurysm, NAFL=non-alcoholic fatty Liver, SNP = single nucleotide polymorphism.
