# Supplementary material for: Causal insights into how NAFLD progression drives abdominal aortic aneurysm: A bidirectional MR study integrating genetic and multi-omics profiling
Source: Medicine (Baltimore). 2026 May 8;105(19):e48613. doi: 10.1097/MD.0000000000048613 (PMC13166516; doi:10.1097/MD.0000000000048613)
Supplement: Supplementary file 5 [file medi-105-e48613-s009.doc]

Table S5. Instrumental variables used in MR analysis of the association between NAFLD/NASH and AAA.

| Exposure | Outcome | SNP | Effect_allele | Other_allele | Exposure | | | Outcome | | | F |
| --- | --- | --- | --- | --- | --- | --- | --- | --- | --- | --- | --- |
|  |  |  |  |  | Beta | SE | pval | Beta | SE | pval |  |
| NAFLD/NASH | AAA | rs112924304 | A | G | 0.53356511073548 | 0.108379102152586 | 8.51680129650672e-07 | 0.020486 | 0.076979 | 0.7901 | 24.23727273 |
| NAFLD/NASH | AAA | rs117792612 | T | C | 0.707050085728937 | 0.148976912755132 | 2.07439780227146e-06 | 0.10771 | 0.083044 | 0.1946 | 22.52487649 |
| NAFLD/NASH | AAA | rs12074944 | T | G | 0.259282597930083 | 0.0547951432055346 | 2.22478805979138e-06 | 0.0091976 | 0.028927 | 0.7505 | 22.39043889 |
| NAFLD/NASH | AAA | rs12077210 | T | C | 0.394741144745189 | 0.0726877488252762 | 5.6151665142359e-08 | 0.030343 | 0.042124 | 0.4713 | 29.49187299 |
| NAFLD/NASH | AAA | rs138270466 | G | A | 0.412109650826833 | 0.0900821341828262 | 4.76627019589598e-06 | 0.015933 | 0.039147 | 0.684 | 20.92898836 |
| NAFLD/NASH | AAA | rs139648192 | T | C | 0.430482871083452 | 0.0790695383043084 | 5.19925691551232e-08 | -0.058744 | 0.046169 | 0.2032 | 29.64103371 |
| NAFLD/NASH | AAA | rs141180697 | T | G | 0.529451087889156 | 0.113582930928004 | 3.1412460268537e-06 | 0.096193 | 0.062836 | 0.1258 | 21.72828993 |
| NAFLD/NASH | AAA | rs1649202 | G | A | 0.173112617708645 | 0.037041964667682 | 2.96226711865817e-06 | 0.01761 | 0.019251 | 0.3603 | 21.8408442 |
| NAFLD/NASH | AAA | rs17216588 | T | C | 0.477475644084437 | 0.0638057770805153 | 7.24480018630236e-14 | -0.0057723 | 0.033209 | 0.862 | 55.99927984 |
| NAFLD/NASH | AAA | rs188987671 | G | A | 0.593326845277734 | 0.115687405083771 | 2.91738442334644e-07 | 0.11057 | 0.065097 | 0.0894 | 26.30363913 |
| NAFLD/NASH | AAA | rs192788238 | T | C | 0.78845736036427 | 0.14959641649115 | 1.36005820277626e-07 | 0.04323 | 0.085835 | 0.6145 | 27.77883593 |
| NAFLD/NASH | AAA | rs2068834 | C | T | 0.263901543786378 | 0.0406519654285864 | 8.4856819612865e-11 | -0.00087318 | 0.020158 | 0.9654 | 42.1425457 |
| NAFLD/NASH | AAA | rs60405540 | T | C | 0.572108852182889 | 0.119991559459719 | 1.8614770194656e-06 | 0.019123 | 0.080776 | 0.8129 | 22.7329575 |
| NAFLD/NASH | AAA | rs62245579 | A | G | 0.423305026236495 | 0.0892293704283797 | 2.0952849103087e-06 | 0.10753 | 0.0444 | 0.01544 | 22.50563095 |
| NAFLD/NASH | AAA | rs7093541 | C | T | 0.38253760346446 | 0.078698346669535 | 1.16907220564259e-06 | 0.059976 | 0.057204 | 0.2944 | 23.62746064 |
| NAFLD/NASH | AAA | rs73459130 | G | A | 0.347129531095201 | 0.0726368196295568 | 1.76191266778872e-06 | -0.044471 | 0.044339 | 0.3159 | 22.83860021 |
| NAFLD/NASH | AAA | rs74714524 | T | C | -0.343899752 | 0.0709096066364773 | 1.2356544413352e-06 | -0.013924 | 0.027896 | 0.6177 | 23.52088036 |
| NAFLD/NASH | AAA | rs7652801 | G | A | 0.422649932862265 | 0.0845153732775202 | 5.70738050818969e-07 | 0.034597 | 0.069565 | 0.619 | 25.00864609 |
| NAFLD/NASH | AAA | rs9467334 | A | C | 0.621651178854875 | 0.13292137691651 | 2.91338292055354e-06 | -0.035213 | 0.10696 | 0.742 | 21.87277373 |
| NAFLD/NASH | AAA | rs9830228 | G | A | -0.522223669 | 0.1140543583622 | 4.67816278640912e-06 | -0.020565 | 0.043839 | 0.639 | 20.96473282 |
| NAFLD/NASH | AAA | rs9925291 | T | C | 0.545806592661236 | 0.114293789069986 | 1.79287771301673e-06 | -0.029525 | 0.10561 | 0.7798 | 22.80511638 |

AAA = abdominal aortic aneurysm, NAFLD = non-alcoholic fatty liver disease, NASH = non-alcoholic steatohepatitis, SNP = single nucleotide polymorphism.
