# Supplementary material for: Causal insights into how NAFLD progression drives abdominal aortic aneurysm: A bidirectional MR study integrating genetic and multi-omics profiling
Source: Medicine (Baltimore). 2026 May 8;105(19):e48613. doi: 10.1097/MD.0000000000048613 (PMC13166516; doi:10.1097/MD.0000000000048613)
Supplement: Supplementary file 6 [file medi-105-e48613-s010.doc]

Table S6. Instrumental variables used in MR analysis of the association between AAA and NAFLD/NASH.

| Exposure | Outcome | SNP | Effect_allele | Other_allele | Exposure | | | Outcome | | | F |
| --- | --- | --- | --- | --- | --- | --- | --- | --- | --- | --- | --- |
|  |  |  |  |  | Beta | SE | pval | Beta | SE | pval |  |
| AAA | NAFLD/NASH | rs10455872 | G | A | 0.3189 | 0.035182 | 1.255e-19 | -0.004912044 | 0.019928770856492 | 0.805310648901951 | 82.16143009 |
| AAA | NAFLD/NASH | rs12532479 | C | T | 0.16069 | 0.027659 | 6.26e-09 | -0.019284761 | 0.0581728985614712 | 0.740261056808956 | 33.75240728 |
| AAA | NAFLD/NASH | rs12740374 | T | G | -0.17961 | 0.021705 | 1.285e-16 | -0.05551271 | 0.0470552043777663 | 0.238105304225788 | 68.47648281 |
| AAA | NAFLD/NASH | rs1537373 | G | T | 0.20234 | 0.017944 | 1.727e-29 | 0.00995033085316809 | 0.0276584529694477 | 0.719028624430525 | 127.1525195 |
| AAA | NAFLD/NASH | rs1806920 | A | G | 0.099259 | 0.018024 | 3.652e-08 | 0.0581589141735321 | 0.0361669499615052 | 0.107820291039988 | 30.32755742 |
| AAA | NAFLD/NASH | rs2227564 | C | T | -0.13589 | 0.020306 | 2.203e-11 | 0.0366639843715915 | 0.0332928216970969 | 0.270784409398792 | 44.7843457 |
| AAA | NAFLD/NASH | rs35247409 | T | C | -0.12817 | 0.022729 | 1.71e-08 | 0.071389996086673 | 0.0473529463822296 | 0.131653178894209 | 31.79890105 |
| AAA | NAFLD/NASH | rs4845373 | T | C | -0.10324 | 0.018448 | 2.192e-08 | 0.118671529717499 | 0.0414291344739202 | 0.00417739017465571 | 31.31824461 |
| AAA | NAFLD/NASH | rs58365910 | C | T | 0.13508 | 0.018956 | 1.032e-12 | -0.091896015 | 0.0545352330410742 | 0.0919739010859515 | 50.77953316 |
| AAA | NAFLD/NASH | rs6590455 | C | T | 0.11442 | 0.020059 | 1.169e-08 | -0.0009995 | 0.00452730367078924 | 0.825270233509679 | 32.53758608 |
| AAA | NAFLD/NASH | rs79973522 | C | T | 0.1861 | 0.031935 | 5.631e-09 | -0.009949331 | 3.70706787222256 | 0.997858574964976 | 33.95931374 |
| AAA | NAFLD/NASH | rs9506822 | G | A | -0.12948 | 0.021753 | 2.642e-09 | -0.041342964 | 0.0684462209115763 | 0.545829595659632 | 35.42966629 |

AAA = abdominal aortic aneurysm, NAFLD = non-alcoholic fatty liver disease, NASH = non-alcoholic steatohepatitis, SNP = single nucleotide polymorphism.
