# Supplementary material for: Causal insights into how NAFLD progression drives abdominal aortic aneurysm: A bidirectional MR study integrating genetic and multi-omics profiling
Source: Medicine (Baltimore). 2026 May 8;105(19):e48613. doi: 10.1097/MD.0000000000048613 (PMC13166516; doi:10.1097/MD.0000000000048613)
Supplement: Supplementary file 7 [file medi-105-e48613-s011.doc]

Table S7. Instrumental variables used in MR analysis of the association between NAFLD/NASH and TAA.

| Exposure | Outcome | SNP | Effect_allele | Other_allele | Exposure | | | Outcome | | | F |
| --- | --- | --- | --- | --- | --- | --- | --- | --- | --- | --- | --- |
|  |  |  |  |  | Beta | SE | pval | Beta | SE | pval |  |
| NAFLD/NASH | TAA | rs10924444 | A | G | 0.538829820175588 | 0.115701029788168 | 3.20714152209264e-06 | 0.02862 | 0.0688311 | 0.677556 | 21.68846403 |
| NAFLD/NASH | TAA | rs112924304 | A | G | 0.53356511073548 | 0.108379102152586 | 8.51680129650672e-07 | -0.0652115 | 0.0602396 | 0.279015 | 24.23727273 |
| NAFLD/NASH | TAA | rs117792612 | T | C | 0.707050085728937 | 0.148976912755132 | 2.07439780227146e-06 | 0.0775861 | 0.0897193 | 0.387168 | 22.52487649 |
| NAFLD/NASH | TAA | rs12074944 | T | G | 0.259282597930083 | 0.0547951432055346 | 2.22478805979138e-06 | -0.00336448 | 0.0319978 | 0.916259 | 22.39043889 |
| NAFLD/NASH | TAA | rs12077210 | T | C | 0.394741144745189 | 0.0726877488252762 | 5.6151665142359e-08 | 0.0262807 | 0.0415972 | 0.527524 | 29.49187299 |
| NAFLD/NASH | TAA | rs138270466 | G | A | 0.412109650826833 | 0.0900821341828262 | 4.76627019589598e-06 | -0.0341972 | 0.0420472 | 0.416044 | 20.92898836 |
| NAFLD/NASH | TAA | rs139648192 | T | C | 0.430482871083452 | 0.0790695383043084 | 5.19925691551232e-08 | 0.0747919 | 0.0519597 | 0.150031 | 29.64103371 |
| NAFLD/NASH | TAA | rs141180697 | T | G | 0.529451087889156 | 0.113582930928004 | 3.1412460268537e-06 | 0.196399 | 0.083813 | 0.0191139 | 21.72828993 |
| NAFLD/NASH | TAA | rs1649202 | G | A | 0.173112617708645 | 0.037041964667682 | 2.96226711865817e-06 | 0.00414193 | 0.0201879 | 0.83744 | 21.8408442 |
| NAFLD/NASH | TAA | rs17216588 | T | C | 0.477475644084437 | 0.0638057770805153 | 7.24480018630236e-14 | 0.0234676 | 0.040273 | 0.560088 | 55.99927984 |
| NAFLD/NASH | TAA | rs188987671 | G | A | 0.593326845277734 | 0.115687405083771 | 2.91738442334644e-07 | -0.0569197 | 0.0946103 | 0.547425 | 26.30363913 |
| NAFLD/NASH | TAA | rs192788238 | T | C | 0.78845736036427 | 0.14959641649115 | 1.36005820277626e-07 | 0.0498091 | 0.0731708 | 0.496046 | 27.77883593 |
| NAFLD/NASH | TAA | rs2068834 | C | T | 0.263901543786378 | 0.0406519654285864 | 8.4856819612865e-11 | -0.0243474 | 0.02205 | 0.269511 | 42.1425457 |
| NAFLD/NASH | TAA | rs4264069 | G | A | 0.543486406005539 | 0.108894555180562 | 6.00855479489387e-07 | -0.0537728 | 0.0602175 | 0.371869 | 24.90950177 |
| NAFLD/NASH | TAA | rs60405540 | T | C | 0.572108852182889 | 0.119991559459719 | 1.8614770194656e-06 | -0.0510918 | 0.10461 | 0.625265 | 22.7329575 |
| NAFLD/NASH | TAA | rs62245579 | A | G | 0.423305026236495 | 0.0892293704283797 | 2.0952849103087e-06 | 0.0266324 | 0.0432205 | 0.537763 | 22.50563095 |
| NAFLD/NASH | TAA | rs7093541 | C | T | 0.38253760346446 | 0.078698346669535 | 1.16907220564259e-06 | 0.125166 | 0.0711716 | 0.0786357 | 23.62746064 |
| NAFLD/NASH | TAA | rs73459130 | G | A | 0.347129531095201 | 0.0726368196295568 | 1.76191266778872e-06 | -0.00983403 | 0.0606898 | 0.871276 | 22.83860021 |
| NAFLD/NASH | TAA | rs74714524 | T | C | -0.343899752 | 0.0709096066364773 | 1.2356544413352e-06 | -0.0344595 | 0.0319769 | 0.281195 | 23.52088042 |
| NAFLD/NASH | TAA | rs7652801 | G | A | 0.422649932862265 | 0.0845153732775202 | 5.70738050818969e-07 | -0.123041 | 0.0985319 | 0.211761 | 25.00864609 |
| NAFLD/NASH | TAA | rs9467334 | A | C | 0.621651178854875 | 0.13292137691651 | 2.91338292055354e-06 | -0.0909788 | 0.174911 | 0.602963 | 21.87277373 |
| NAFLD/NASH | TAA | rs9830228 | G | A | -0.522223669 | 0.1140543583622 | 4.67816278640912e-06 | -0.124521 | 0.0744967 | 0.0946237 | 20.9647328 |
| NAFLD/NASH | TAA | rs9925291 | T | C | 0.545806592661236 | 0.114293789069986 | 1.79287771301673e-06 | -0.0216183 | 0.231984 | 0.925753 | 22.80511638 |

AAA = abdominal aortic aneurysm, NAFLD = non-alcoholic fatty liver disease, NASH = non-alcoholic steatohepatitis, SNP = single nucleotide polymorphism.
