# Supplementary material for: Causal insights into how NAFLD progression drives abdominal aortic aneurysm: A bidirectional MR study integrating genetic and multi-omics profiling
Source: Medicine (Baltimore). 2026 May 8;105(19):e48613. doi: 10.1097/MD.0000000000048613 (PMC13166516; doi:10.1097/MD.0000000000048613)
Supplement: Supplementary file 9 [file medi-105-e48613-s013.doc]

Table S9. Instrumental variables used in MR analysis of the association between Metabolism and NAFLD/NASH.

| Exposure | Outcome | SNP | Effect_allele | Other_allele | Exposure | | | Outcome | | | F |
| --- | --- | --- | --- | --- | --- | --- | --- | --- | --- | --- | --- |
|  |  |  |  |  | Beta | SE | pval | Beta | SE | pval |  |
| 3-Hydroxybutyrate levels | NAFLD/NASH | rs1009062 | T | G | 0.029310529 | 0.005344012 | 4.141E-08 | -0.018821754 | 0.025828994 | 0.466181245 | 30.08195869 |
| 3-Hydroxybutyrate levels | NAFLD/NASH | rs10800459 | T | C | 0.023381531 | 0.005063077 | 0.000003874 | 0.01572296 | 0.079561488 | 0.843342197 | 21.32605333 |
| 3-Hydroxybutyrate levels | NAFLD/NASH | rs11046944 | A | C | 0.026955328 | 0.006073888 | 0.000009084 | -0.011928571 | 0.028624042 | 0.676874016 | 19.69469928 |
| 3-Hydroxybutyrate levels | NAFLD/NASH | rs111962457 | T | C | -0.048803463 | 0.010139498 | 0.000001485 | -0.056380333 | 0.107713478 | 0.600675906 | 23.16658324 |
| 3-Hydroxybutyrate levels | NAFLD/NASH | rs117249464 | T | C | 0.050156458 | 0.0109973 | 0.000005096 | -0.071173819 | 0.068722306 | 0.30035484 | 20.80056769 |
| 3-Hydroxybutyrate levels | NAFLD/NASH | rs117643180 | A | C | -0.11611736 | 0.018814012 | 6.749E-10 | 0.094400675 | 0.182088262 | 0.604155767 | 38.09123795 |
| 3-Hydroxybutyrate levels | NAFLD/NASH | rs1215112 | A | G | 0.035347868 | 0.007748182 | 0.000005065 | 0.050767117 | 0.064816988 | 0.433487462 | 20.81231504 |
| 3-Hydroxybutyrate levels | NAFLD/NASH | rs12653566 | A | G | 0.038439582 | 0.008150244 | 0.000002401 | 0.030562306 | 0.035758562 | 0.392725568 | 22.24383871 |
| 3-Hydroxybutyrate levels | NAFLD/NASH | rs12867528 | T | G | 0.046568282 | 0.009473731 | 8.855E-07 | -0.102032726 | 0.086539849 | 0.238387851 | 24.16194969 |
| 3-Hydroxybutyrate levels | NAFLD/NASH | rs13284054 | T | C | 0.058332592 | 0.008529637 | 7.985E-12 | -0.019802627 | 0.042871097 | 0.644145211 | 46.76867161 |
| 3-Hydroxybutyrate levels | NAFLD/NASH | rs147507218 | T | C | -0.090779907 | 0.020189685 | 0.000006913 | 0.189346172 | 0.178529336 | 0.288876924 | 20.21687355 |
| 3-Hydroxybutyrate levels | NAFLD/NASH | rs148921528 | T | C | -0.095815733 | 0.021411378 | 0.000007641 | 0.21927604 | 0.132307922 | 0.097455598 | 20.02525382 |
| 3-Hydroxybutyrate levels | NAFLD/NASH | rs1698148 | A | G | 0.024323562 | 0.005313447 | 0.0000047 | -0.037295785 | 0.040813667 | 0.360818656 | 20.95536946 |
| 3-Hydroxybutyrate levels | NAFLD/NASH | rs189288878 | A | G | 0.12193958 | 0.025917344 | 0.000002539 | 0.177692288 | 0.285544331 | 0.533749108 | 22.13614635 |
| 3-Hydroxybutyrate levels | NAFLD/NASH | rs1982099 | A | C | 0.025819443 | 0.00575186 | 0.00000716 | 0.006521217 | 0.09263381 | 0.943877037 | 20.14979994 |
| 3-Hydroxybutyrate levels | NAFLD/NASH | rs2760105 | A | G | -0.023718773 | 0.005329113 | 0.000008555 | 0.028605257 | 0.058972262 | 0.627631722 | 19.80926027 |
| 3-Hydroxybutyrate levels | NAFLD/NASH | rs277407 | A | C | 0.040112082 | 0.006108676 | 5.154E-11 | -0.067658648 | 0.040736964 | 0.096740314 | 43.11713661 |
| 3-Hydroxybutyrate levels | NAFLD/NASH | rs28442086 | A | G | 0.025072108 | 0.005626609 | 0.000008351 | 0.094400675 | 0.042780984 | 0.027341761 | 19.85554772 |
| 3-Hydroxybutyrate levels | NAFLD/NASH | rs28929474 | T | C | -0.12834066 | 0.02173188 | 3.513E-09 | 0.37569295 | 0.158269259 | 0.017608078 | 34.87607109 |
| 3-Hydroxybutyrate levels | NAFLD/NASH | rs36039532 | T | C | -0.073759532 | 0.015999023 | 0.000004022 | 0.102556588 | 0.111014963 | 0.355585928 | 21.25411341 |
| 3-Hydroxybutyrate levels | NAFLD/NASH | rs45611540 | A | G | 0.098509068 | 0.020913955 | 0.000002475 | 0.064850972 | 0.10535911 | 0.538209278 | 22.1857286 |
| 3-Hydroxybutyrate levels | NAFLD/NASH | rs4762701 | A | G | -0.03592502 | 0.007591931 | 0.000002223 | 0.060812139 | 0.058830925 | 0.301287468 | 22.39150082 |
| 3-Hydroxybutyrate levels | NAFLD/NASH | rs4841132 | A | G | -0.1021712 | 0.00837497 | 3.124E-34 | 0.136277618 | 0.07719206 | 0.077490568 | 148.8278034 |
| 3-Hydroxybutyrate levels | NAFLD/NASH | rs61824906 | A | G | 0.061211762 | 0.013833861 | 0.000009654 | -0.106160196 | 0.078828798 | 0.178070929 | 19.57837131 |
| 3-Hydroxybutyrate levels | NAFLD/NASH | rs62111729 | A | G | 0.066828006 | 0.015124728 | 0.000009941 | 0.070458464 | 0.100578774 | 0.483596283 | 19.52250189 |
| 3-Hydroxybutyrate levels | NAFLD/NASH | rs627108 | T | C | 0.033980132 | 0.005347155 | 2.087E-10 | -0.051819749 | 0.045384688 | 0.253541635 | 40.3829669 |
| 3-Hydroxybutyrate levels | NAFLD/NASH | rs6562430 | A | C | -0.026383252 | 0.005484005 | 0.000001502 | 0.037182791 | 0.038312231 | 0.331787294 | 23.14485954 |
| 3-Hydroxybutyrate levels | NAFLD/NASH | rs7024300 | T | C | 0.086831714 | 0.015874605 | 4.504E-08 | -0.095080206 | 0.12887561 | 0.46065589 | 29.91882311 |
| 3-Hydroxybutyrate levels | NAFLD/NASH | rs7424006 | A | G | 0.047170042 | 0.01009582 | 0.000002979 | -0.037295785 | 0.065119452 | 0.566828344 | 21.82945747 |
| 3-Hydroxybutyrate levels | NAFLD/NASH | rs77807654 | A | C | -0.08142796 | 0.017608537 | 0.000003758 | 0.129272336 | 0.220031911 | 0.556857002 | 21.38425747 |

NAFLD = non-alcoholic fatty liver disease, NASH = non-alcoholic steatohepatitis, SNP = single nucleotide polymorphism.
