# Supplementary material for: Causal insights into how NAFLD progression drives abdominal aortic aneurysm: A bidirectional MR study integrating genetic and multi-omics profiling
Source: Medicine (Baltimore). 2026 May 8;105(19):e48613. doi: 10.1097/MD.0000000000048613 (PMC13166516; doi:10.1097/MD.0000000000048613)
Supplement: Supplementary file 10 [file medi-105-e48613-s014.doc]

Table S10. Instrumental variables used in MR analysis of the association between Liposome and NAFLD/NASH.

| Exposure | Outcome | SNP | Effect_allele | Other_allele | Exposure | | | Outcome | | | F |
| --- | --- | --- | --- | --- | --- | --- | --- | --- | --- | --- | --- |
|  |  |  |  |  | Beta | SE | pval | Beta | SE | pval |  |
| Phosphatidylcholine (18:1_20:4) levels | NAFLD/NASH | rs1077834 | C | T | 0.0986416 | 0.0192062 | 2.88092E-07 | 0.025667747 | 0.051156406 | 0.615843112 | 26.37036942 |
| Phosphatidylcholine (18:1_20:4) levels | NAFLD/NASH | rs10889353 | C | A | -0.0853107 | 0.0188257 | 5.94617E-06 | -0.011667805 | 0.015861382 | 0.461967552 | 20.5297488 |
| Phosphatidylcholine (18:1_20:4) levels | NAFLD/NASH | rs113394924 | G | T | 0.18654 | 0.0296908 | 3.52494E-10 | -0.103695236 | 0.092619364 | 0.262890717 | 39.46199502 |
| Phosphatidylcholine (18:1_20:4) levels | NAFLD/NASH | rs113913626 | T | C | -0.212622 | 0.0481015 | 9.99307E-06 | 0.076034686 | 0.282462533 | 0.787787297 | 19.5334093 |
| Phosphatidylcholine (18:1_20:4) levels | NAFLD/NASH | rs116814948 | C | T | -0.425137 | 0.0940047 | 6.20112E-06 | -0.056993722 | 0.144114587 | 0.692492341 | 20.44737506 |
| Phosphatidylcholine (18:1_20:4) levels | NAFLD/NASH | rs147549994 | T | G | 0.232307 | 0.0453863 | 3.15693E-07 | 0.088926209 | 0.122683196 | 0.468547585 | 26.19111127 |
| Phosphatidylcholine (18:1_20:4) levels | NAFLD/NASH | rs147797702 | C | T | -0.489187 | 0.0890169 | 4.02686E-08 | -0.17578298 | 0.329319611 | 0.593496362 | 30.1914363 |
| Phosphatidylcholine (18:1_20:4) levels | NAFLD/NASH | rs173539 | T | C | 0.0848865 | 0.018448 | 4.26473E-06 | 0.060153923 | 0.045361202 | 0.184803412 | 21.16691503 |
| Phosphatidylcholine (18:1_20:4) levels | NAFLD/NASH | rs2943813 | C | T | -0.129638 | 0.0204126 | 2.26383E-10 | -0.057325067 | 0.036069335 | 0.111992226 | 40.32244754 |
| Phosphatidylcholine (18:1_20:4) levels | NAFLD/NASH | rs3018617 | G | A | -0.0918283 | 0.0188177 | 1.08226E-06 | -0.072678225 | 0.04519337 | 0.107799872 | 23.80668356 |
| Phosphatidylcholine (18:1_20:4) levels | NAFLD/NASH | rs4846905 | G | A | 0.0941932 | 0.0195614 | 1.49895E-06 | 0.053084371 | 0.05148161 | 0.302478601 | 23.18025059 |
| Phosphatidylcholine (18:1_20:4) levels | NAFLD/NASH | rs531117 | T | C | -0.113887 | 0.0198453 | 9.95537E-09 | 0.022739487 | 0.097378734 | 0.81536079 | 32.92394448 |
| Phosphatidylcholine (18:1_20:4) levels | NAFLD/NASH | rs6083838 | G | A | -0.0745078 | 0.0168167 | 9.53395E-06 | -0.07881118 | 0.041960284 | 0.060349329 | 19.62460023 |
| Phosphatidylcholine (18:1_20:4) levels | NAFLD/NASH | rs61978272 | T | C | -0.0981277 | 0.0186254 | 1.41417E-07 | -0.014098924 | 0.073880117 | 0.848654736 | 27.74922855 |
| Phosphatidylcholine (18:1_20:4) levels | NAFLD/NASH | rs641738 | C | T | -0.0851942 | 0.0170472 | 5.94032E-07 | -0.055434707 | 0.045940111 | 0.22755797 | 24.96852211 |
| Phosphatidylcholine (18:1_20:4) levels | NAFLD/NASH | rs6850591 | C | T | -0.0807281 | 0.0168282 | 1.64001E-06 | 0.09036244 | 0.042402364 | 0.033083309 | 23.00663085 |
| Phosphatidylcholine (18:1_20:4) levels | NAFLD/NASH | rs73176681 | G | A | 0.184219 | 0.0403803 | 5.15201E-06 | 0.082501222 | 0.098298789 | 0.401306418 | 20.80696087 |
| Phosphatidylcholine (18:1_20:4) levels | NAFLD/NASH | rs73618588 | A | G | -0.130098 | 0.0287245 | 6.01423E-06 | -0.054350595 | 0.068345787 | 0.426479942 | 20.50761599 |
| Phosphatidylcholine (18:1_20:4) levels | NAFLD/NASH | rs75976069 | C | T | -0.260712 | 0.053834 | 1.30559E-06 | -0.384780517 | 0.21051472 | 0.067578353 | 23.44701892 |
| Phosphatidylcholine (18:1_20:4) levels | NAFLD/NASH | rs76582138 | G | A | -0.45817 | 0.101575 | 6.5598E-06 | -0.135247149 | 0.201949947 | 0.503045041 | 20.34035498 |
| Phosphatidylcholine (18:1_20:4) levels | NAFLD/NASH | rs79113397 | C | T | 0.0995155 | 0.0201051 | 7.58983E-07 | -0.075046874 | 0.071239642 | 0.292138184 | 24.49333234 |
| Sphingomyelin (d36:2) levels | NAFLD/NASH | rs117546336 | G | T | -0.189726 | 0.0398834 | 2.00297E-06 | -0.015316704 | 0.07337588 | 0.834648841 | 22.62289837 |
| Sphingomyelin (d36:2) levels | NAFLD/NASH | rs12207488 | A | G | -0.0963826 | 0.0215973 | 8.21432E-06 | -0.045101974 | 0.06206952 | 0.467448709 | 19.91027438 |
| Sphingomyelin (d36:2) levels | NAFLD/NASH | rs13005731 | G | T | -0.504392 | 0.112784 | 7.85598E-06 | -0.058264908 | 0.17614603 | 0.740813566 | 19.99494826 |
| Sphingomyelin (d36:2) levels | NAFLD/NASH | rs1452773 | G | T | -0.10869 | 0.0239916 | 5.98003E-06 | -0.065605757 | 0.066774022 | 0.325851516 | 20.51821825 |
| Sphingomyelin (d36:2) levels | NAFLD/NASH | rs146465816 | A | G | -0.360803 | 0.0807842 | 8.07216E-06 | 0.035367144 | 0.113596925 | 0.755542983 | 19.94188991 |
| Sphingomyelin (d36:2) levels | NAFLD/NASH | rs148860058 | A | G | 0.400771 | 0.0871824 | 4.36666E-06 | 0.122217633 | 0.150398676 | 0.416433443 | 21.12583228 |
| Sphingomyelin (d36:2) levels | NAFLD/NASH | rs17276940 | T | C | -0.218629 | 0.046071 | 2.11921E-06 | -0.215919706 | 0.171601936 | 0.208298049 | 22.51329958 |
| Sphingomyelin (d36:2) levels | NAFLD/NASH | rs174544 | A | C | -0.178175 | 0.0171876 | 5.30012E-25 | -0.048875168 | 0.0425215 | 0.250381816 | 107.4340507 |
| Sphingomyelin (d36:2) levels | NAFLD/NASH | rs313950 | A | G | -0.076451 | 0.0170369 | 7.31829E-06 | 0.015873349 | 0.048321695 | 0.742538768 | 20.13094137 |
| Sphingomyelin (d36:2) levels | NAFLD/NASH | rs6444176 | T | C | 0.175355 | 0.0376277 | 3.21656E-06 | -0.052662653 | 0.600489495 | 0.930115485 | 21.71200116 |
| Sphingomyelin (d36:2) levels | NAFLD/NASH | rs68137603 | T | C | 0.153224 | 0.0346304 | 9.79641E-06 | 0.01093994 | 0.031152999 | 0.725462223 | 19.57120054 |
| Sphingomyelin (d36:2) levels | NAFLD/NASH | rs7253584 | C | T | 0.180906 | 0.0177097 | 2.47575E-24 | 0.043220702 | 0.043936148 | 0.325255054 | 104.3187622 |
| Sphingomyelin (d36:2) levels | NAFLD/NASH | rs72879665 | A | G | 0.381868 | 0.0833797 | 4.72775E-06 | 0.167207919 | 0.19684115 | 0.395627538 | 20.96934034 |
| Sphingomyelin (d36:2) levels | NAFLD/NASH | rs7700432 | A | G | -0.0794721 | 0.0173224 | 4.55137E-06 | -0.076017486 | 0.041449244 | 0.066655521 | 21.04224845 |
| Sphingomyelin (d36:2) levels | NAFLD/NASH | rs77645768 | A | G | 0.242701 | 0.0537632 | 6.44612E-06 | 0.178982656 | 0.140429007 | 0.202471427 | 20.37285072 |
| Sphingomyelin (d36:2) levels | NAFLD/NASH | rs78999781 | C | T | -0.147582 | 0.0290763 | 3.95488E-07 | -0.056147161 | 0.065404337 | 0.390637215 | 25.75534623 |
| Sphingomyelin (d36:2) levels | NAFLD/NASH | rs7920200 | A | G | -0.188214 | 0.042306 | 8.75491E-06 | -0.159934038 | 0.076899542 | 0.037545826 | 19.78694792 |
| Sphingomyelin (d36:2) levels | NAFLD/NASH | rs9738226 | G | A | 0.100806 | 0.0168639 | 2.37014E-09 | -0.044973366 | 0.035479869 | 0.20495006 | 35.72198794 |

NAFLD = non-alcoholic fatty liver disease, NASH = non-alcoholic steatohepatitis, SNP = single nucleotide polymorphism.
